# Trends and determinants of an acceptable antenatal care coverage in Ethiopia, evidence from 2005-2016 Ethiopian demographic and health survey; Multivariate decomposition analysis

**DOI:** 10.1186/s13690-020-00510-2

**Published:** 2020-12-04

**Authors:** Tilahun Yemanu Birhan, Wullo Sisay Seretew

**Affiliations:** Department of Epidemiology and Biostatistics, Institute of Public Health, College of Medicine and Health Science, University of Gondar, Gondar, Ethiopia

**Keywords:** ANC, Ethiopia, Multivariate decomposition analysis, Trend, And women

## Abstract

**Background:**

an acceptable antenatal care (ANC4+) is defined as attending at least four antenatal care visit, received at least one dose of tetanus toxoid (TT) injections and consumed 100 iron-folic acids (IFA) tablets/syrup during the last pregnancy. Since maternal health care service utilization continues to be an essential indicator for monitoring the improvements of maternal and child health outcomes. This study aimed to analyze the trends and determinants that contributed to the change in an acceptable antenatal care visit over the last 10 years in Ethiopia.

**Methods:**

Nationally representative repeated cross-sectional survey was conducted using 2005, 2011, and 2016 Ethiopian Demographic and Health Survey datasets. The data were weighted and analyzed by STATA 14.1 software. Multivariate decomposition regression analysis was used to identify factors that contribute for the change in an acceptable antenatal care visit. A *p*-value < 0.05 was taken to declare statistically significant predictors to acceptable antenatal care visit.

**Results:**

among the reproductive age women the rate of an acceptable antenatal care visits was increased from 16% in 2005 to 35% in 2016 in Ethiopia. In the multivariate decomposition analysis, about 29% of the increase in acceptable antenatal care visit was due to a difference in composition of women (endowments) across the surveys. Residence, religion, husband educational attainment, and wealth status was the main source of compositional change factors for the improvements of an acceptable antenatal care visit. Almost two-thirds of an overall change in acceptable antenatal care visit was due to the difference in coefficients/ change in behavior of the population. Religion, educational attainment (both women and husband), and residence are significantly contributed to the change in full antenatal care visit in Ethiopia over the last decades.

**Conclusion:**

Besides the relevance of receiving an acceptable antenatal care visit for pregnant women and their babies, an acceptable antenatal care visit was slightly increased over time in Ethiopia. Women’s characteristics and behavior change were significantly associated with the change in acceptable antenatal care visits. Public interventions needed to improve acceptable antenatal care coverage, women’s education, and further advancing of health care facilities in rural communities should be done to maintain the further improvements acceptable antenatal care visits.

## Background

An acceptable antenatal care (ANC4+) is defined as attending at least four antenatal care visit, received at least one dose of tetanus toxoid (TT) injections and consumed 100 iron-folic acids (IFA) tablets/syrup during the last pregnancy [1]. Globally, antenatal care (ANC) remains an essential intervention for improving maternal and child health. World Health Organization (WHO) had previously recommended at least four visits (i.e. the `reduced’ ANC model) and more recently, the standard ANC model has been implemented at least eight ANC contacts [2–4]. Globally, 72.9% (95% CI 69.1–76.8) of women used ANC including blood pressure monitoring, urine and blood testing [5, 6], among that only 53.3% (44.3–63.3) was in low-income countries and 74.8% (68.6–80.9) was in lower-middle-income countries while developed countries were used 93.3% (91.4–95.2) [5, 6]. Even though there is high ANC coverage across all low-income countries, nearly a third of women who accessed antenatal care was not received a basic package of three services during their pregnancy [2, 5, 6]. Since, maternal health care service utilization continues to be an essential indicator for monitoring the improvements in maternal and child health outcomes. However, inadequate access to ANC, intrapartum, and postnatal care services are the pertinent reason for high maternal and child morbidities and mortalities in Sub-Saharan Africa (SSA) [7, 8]. ANC performs a vital heroine in ensuring a healthy baby and mother throughout pregnancy and after delivery; all pregnant women receive quality ANC service regardless of their economic, cultural, and social background [9–11]. ANC is very relevant to optimize quality health outcomes, such as normal birth weight, reduction in maternal and child death as well as low postpartum anemia [11, 12]. Despite, progress in reducing maternal mortality and improving the uptake of ANC4+ and tetanus toxoid (TT) injection in Ethiopia, an inclusive understanding of full ANC utilization coverage is still lacking in Ethiopia [7, 13, 14]. Ethiopia is one of the Sub-Saharan countries with the highest maternal mortality (420/100,000 live births) in the world linked with low utilization of full ANC visits and skilled delivery [15, 16]. ANC is one of the main vital indicators for safe motherhood initiative, which helps to reduce pregnancy-related complications and death in developing countries including Ethiopia [4, 11, 17, 18]. Efforts have been made to ensure quality maternal health service across all aspects of populations, national subnational and global levels. However, most health care systems are not accessible to every community in Ethiopia, benefiting the urban than the rural and underprivileged [13, 19–21]. 2016 WHO guideline-recommended eight ANC contacts, five contacts in the third trimester to reduce pregnancy-related complications, morbidity, and mortality, but four visits are still lagging in Ethiopia [1, 4, 22]. An astonishing progress has been done to optimize the coverage of ANC in Ethiopia. However, several factors hindering the availability of ANC services such as lack of improved transportation, inaccessibility to communication technology, low rate of education and low socioeconomic status [19, 23]. This paper aimed to quantify the contributing factors that explain an acceptable ANC coverage, which may be useful for informing policy and indicate specific programming intervention to resolve the utilization of an acceptable ANC visit and further improvements of maternal health service in Ethiopia.

## Methods and materials

### Study design and sampling

This study was based on a secondary analysis of cross-sectional population data from Ethiopia Demographic Health Surveys (EDHS) 20,005, 2011, and 2016 to investigate trends and the factors associated with ANC4+ in Ethiopia.

So far, in Ethiopia, four consecutive surveys were conducted in the cross-sectional years of 2000, 2005, 2011, and 2016 respectively. Similar to other demographic and health surveys, the principal objective Ethiopian Demographic and Health Survey (EDHS) was to offer current and consistent data on fertility and family planning behavior, child mortality, adult and maternal mortality, children’s nutritional status, use of maternal and child health services, as well as data, were collected on knowledge and attitudes of women and men about sexually transmitted diseases and HIV/AIDS and evaluated potential exposure to the risk of HIV infection by exploring high-risk behaviors and condom use.

The sampling frame used for the 2016 EDHS was the Ethiopia Population and Housing Census (EPHC), which was conducted in 2007 by the Ethiopia Central Statistical Agency. The census frame is a complete list of 84,915 *enumeration areas* (EAs) created for the 2007 PHC. An EA is a geographic area covering on average 181 households. The sampling frame contains information about the EA location, type of residence (urban or rural), and an estimated number of residential households. Except for EAs in six zones of the Somali region, each EA has accompanying cartographic materials. These materials delineate geographic locations; boundaries, main access, and landmarks in or outside the EA that help identify the EA. In Somali, a cartographic frame was used in three zones where sketch maps delineating the EA geographic boundaries were available for each EA; in the remaining six zones, satellite image maps were used to provide a map for each EA.

### Variables and measurement

The outcome variable was ‘ANC4+’. A woman was counted as having acceptable ANC, if she had to get four ANC visits, received at least one dose of tetanus toxoid (TT) injections and consumed 100 iron-folic acids (IFA) tablets/syrup during the last pregnancy.

The predictor variables are **Socio-demographic Characteristics:** Age, Marital status, Level of education, media exposure, and occupation

**Socio-cultural factors:** Unplanned Pregnancy, Fear of testing for HIV status, knowledge about ANC benefits, Peer influence, TBA influence, decision-making authority

**Obstetric factors and Economic factors:** Gravida**,** Parity**,** Complications during pregnancy, history of abortion, history of stillbirth, trimester of pregnancy and wealth status

### Data management and analysis

The data were cleaned and analyzed using STATA14 software and the data was weighted for analysis.

The trend was assessed using descriptive analysis by selected explanatory variables of the study population as well as the trend was assessed separately from 2005 to 2011, 2011–2016, and 2005–2016.

Multivariate decomposition analysis of change in ANC4+ was employed to answer the major factors contributing to the difference in the percentage of ANC4+ over the study period. This methods are used for many purposes in economic, demography, and other specialties. The present analysis focused on how the ANC4+ rate responds to difference in women’s characteristics and how these factors shape the differences across surveys conducted at different times. The analysis was a regression analysis of the difference in the percentage of ANC4+ rate between EDHS 2005 and 2016. The multivariate decomposition analysis was to identify the source of difference in the percentage of ANC4+ in the last 10 years. Both the difference in composition (Endowment) of the population and the difference in the effect of characteristics (Coefficients) between the surveys is essential to identify the factors contributing to the increase in ANC4+ rate overtime.

The multivariate decomposition analysis for nonlinear response model utilizes the output from a logistic regression model since it is “a binary outcome” to parcel out the observed difference in ANC4+ into components. The difference in the rate of ANC4+ between the surveys can be attributed to the compositional difference in population (difference characteristics or endowment) and the difference in the effect of explanatory variable (difference in coefficients) between the surveys.

Logit based decomposition analysis technique was used for the analysis of factors contributing to the change in ANC4+ rate over time to identify factors contributing to the ANC4+ in the last 10 years. The change of ANC4+ over time can be attributed to the compositional difference between the surveys and difference in the effect of selected covariates. Hence, the observed difference in ANC4+ between the surveys is additively decomposed into characteristics (or endowments) component and a coefficient (or effect of characteristics) component. For the decomposition analysis, the 2005 EDHS data appended to the 2016 EDHS data by using the command “append”. Since all variables are coded before merging in similar situation.

The mean difference in Y between groups A and B can be decomposed as:

$$ {Y}_A-{Y}_B=F\left({X}_A{\beta}_A\right)-F\left({X}_B{\beta}_B\right) $$

For our logistic regression, the logit or log-odds of ANC4+ is taken as:

$$ Logit(A)- Logit(B)=F\left({X}_A{\beta}_A\right)-F\left({X}_B{\beta}_B\right)=\underset{E}{\underbrace{\left[\mathrm{F}\left(\mathrm{XA}\upbeta \mathrm{A}\right)-\mathrm{F}\left(\mathrm{XB}\upbeta \mathrm{A}\right)\right]}}+\underset{C}{\underbrace{\left[\mathrm{F}\left(\mathrm{XB}\upbeta \mathrm{A}\right)-\mathrm{F}\right(\mathrm{XB}\upbeta \mathrm{B}\Big]}} $$

The *E* component refers to the part of the differential owing to differences in endowments or characteristics. The *C* component refers to that part of the differential attributable to differences in coefficients or effects [24].

The equation can be presented as:

$$ \mathrm{Logit}\ \left(\mathrm{A}\right)-\mathrm{Logit}\ \left(\mathrm{B}\right)=\left[\upbeta 0\mathrm{A}-\upbeta 0\mathrm{B}\right]+\Sigma \mathrm{XijB}\ast \left[\upbeta \mathrm{ijA}-\upbeta \mathrm{ijB}\right]+\Sigma \upbeta \mathrm{ijB}\ast \left[\mathrm{XijA}-\mathrm{XijB}\right] $$

- *X*_*ij*B_ is the proportion of the j^th^ category of the i^th^ determinant in the DHS 2005,
- *X*_*ij*A_ is the proportion of the j^th^ category of the i^th^ determinant in DHS 2016,
- *β*_*ijB*_ is the coefficient of the j^th^ category of the i^th^ determinant in DHS 2005,
- *β*_*ij*A_ is the coefficient of the j^th^ category of the i^th^ determinant in DHS 2016,
- *β*_0B_ is the intercept in the regression equation fitted to DHS 2005, and
- *β*_*0A*_ is the intercept in the regression equation fitted to DHS 2016

The recently developed multivariate decomposition for the non-linear model was used for the decomposition analysis of ANC4+ using mvdcmp STATA command [24].

## Result

### Characteristics of the study population

This section presents the characteristics of respondents over three EDHS surveys. Among the respondents, more women Visit ANC in the second trimester in three consecutive EDHS surveys, and some percentage of women visit in the third trimester. Regarding the husband’s educational status, in 2005 and 2011, 21% of respondents take primary school, increasing to 31% in 2011. In terms of women’s educational status, in 2005 81% were not educated and in 2011 76% were not educated. Similarly, more women were taken primary school in three survey periods. Regarding women’s fertility preference, the difference was not observed between 2011 and 2016 in the category of wants soon. The percentage of women exposed to media about ANC visits increases from 38% in 2005 and 68% in 2016 (Table 1).

**Table 1:** Percentage Distribution of Socio-demographic Characteristics among Respondents from 2005 to 2016 Ethiopian Demographic and Health Survey

Characteristics	Weighted frequency(%) 2005 *N* = 39,246	Weighted frequency(%) 2011 *N* = 44, 691	Weighted frequency(%) 2016 *N* = 41, 392
Trimester of ANC visit			
1st trimester	27.6	31.9	37.1
2nd trimester	53.7	54.8	53.6
3rd trimester	18.7	13.3	9.23
History of abortion			
Yes	4.9	8.4	4.0
No	95.1	91.6	96.0
Region			
Tigray	10.1	11.1	10.7
Afar	6.0	9.7	8.4
Amhara	16.4	13.8	11.4
Oromia	18.8	14.8	14.7
Somali	5.5	7.1	12.2
Benishangul-Gumuz	6.8	7.1	8.7
SNNPR	17.6	8.6	13.6
Gambela	4.9	14.4	6.2
Harari	4.1	5.0	4.8
Addis-Abeba	5.4	3.5	3.9
Dire-Dawa	4.4	5.1	5.3
Religion			
Orthodox	43.7	35.6	33.1
Protestant	16.8	19.0	18.5
Muslim	36.8	43.3	47.1
Catholic	1.1	1.2	0.6
Traditional	1.6	0.9	0.9
Husband educational status			
None	66.5	59.7	56.3
Primary	21.0	31.9	30.6
Secondary+	12.5	8.5	13.2
Women educational status			
None	81.8	76.0	73.2
Primary	12.1	20.2	20.0
Secondary+	6.1	3.8	6.8
Wealth index			
Poor	42.8	48.3	50.4
Middle	17.4	16.8	14.8
Rich	39.8	34.9	34.9
Residence			
Rural	83.9	83.2	81.9
Urban	16.1	16.8	18.1
History of stillbirth			
Yes	95.1	91.6	96.0
No	5.0	8.4	4.0
Place of delivery			
Home	94.7	90.4	73.3
H institution	5.3	9.6	26.7
Media exposure on ANC visit			
Exposed	37.7	52.9	67.9
Non-exposed	62.3	47.1	32.1
Pregnancy complication			
Yes	36.4	23.9	46.7
No	63.6	76.1	53.3
Birth order			
1	13.2	21.0	48.3
2–3	5.9	11.0	29.1
4–5	2.6	6.1	18.2
6+	2.1	4.1	14.7
Anemic level of respondents			
Anemic	30.6	23.4	32.4
Non-anemic	69.4	76.7	67.6
Parity			
1	3.9	21.9	4.2
2–3	16.1	32.2	17.3
4–5	24.2	20.2	24.1
6+	55.6	25.7	54.3
Fertility preference			
Wants soon	38.5	44.3	45.1
Wants later	4.8	4.3	5.0
Undecided	1.4	3.8	45.8
No more wants	55.3	47.6	4.1
Mothers age at birth			
> 20	6.3	9.6	31.4
20–34	5.5	10.7	26.4
35–49	3.5	6.4	21.2
Husband/partner occupational status			
Working	99.3	98.0	89.3
Not working	0.7	2.0	10.7
Women occupational status			
Working	29.2	53.5	43.5
Not working	70.8	46.5	56.5

### Trends of an acceptable antenatal care coverage in Ethiopia

**Fig. 1:**
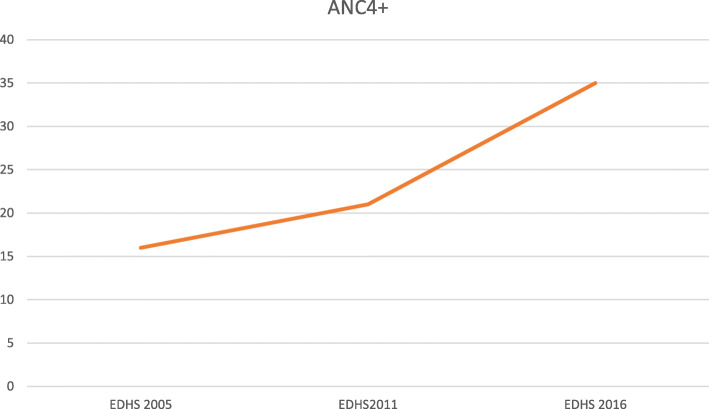
Trends of an acceptable antenatal care (ANC4+) visit over last 10 years in Ethiopia, Ethiopian Demographic and Health Survey 2005–2016

In this section, we present trends of full ANC4+ coverage during three consecutive EDHS survey periods. Perceiving at the overall trend, Ethiopia shows slow progress on the coverage of ANC4+ over a study period, overall trends of full antenatal care coverage was increased from 16% in 2005 to 21% in 2011 and 35% in 2016 (Fig. 1).

**Fig. 2:**
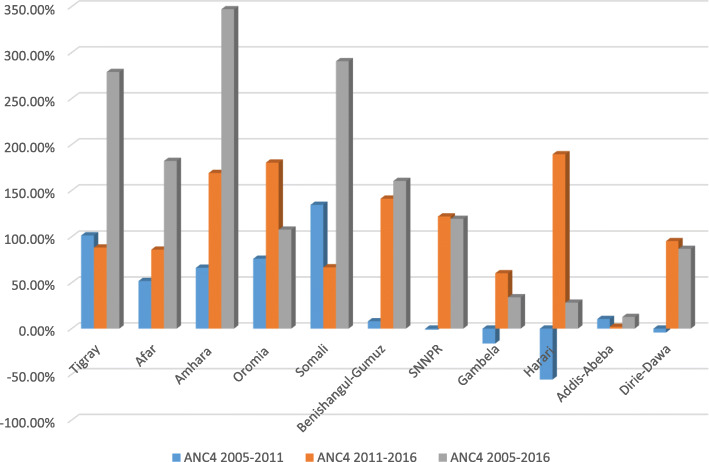
Trends of an acceptable antenatal care (ANC4+) visit across regions over the last 10 years in Ethiopia, Ethiopian demographic and Health Survey 2005–2016

The trends in ANC4+ has increased in Amhara, Somali, Tigray, Afar, SNNPR, Benshangul-Gumuz, and Oromia regions over time (Fig. 2). In terms of residence, the percentage of acceptable ANC visitors increases at a 211 percentage points among rural residents from 2005 to 2016. Regarding maternal education there was an increase in ANC4+ visit among all categories with the highest increase in secondary and higher education from (2011–2016) at 75 percentage points. Similarly in birth order, there was an increase in ANC4+ visit in each category with the highest increase in 6+ birth orders in the entire study period at 145.4% (Table 2).

**Table 2:** Trends of an acceptable antenatal care visit (ANC4+) in Ethiopia by selected characteristics of respondents from 2005 to 2016 Ethiopian Demographic and Health Survey

Characteristics	2005 ***N*** = 39,246	2011 ***N*** = 44, 691	2016 *N* = 42,164	The percentage point difference for ANC4+		
				Phase I 2005–2011(%)	Phase II 2011–2016(%)	Phase III 2005–2016(%)
**Religion**						
Orthodox	21.1	30.4	48.3	44.1	58.9	128.9
Protestant	16.4	17.2	33.5	4.9	94.7	104.3
Muslim	12.6	16.8	27.5	33.3	63.7	118.3
Catholic	11.5	20.8	31.8	80.9	52.9	176.5
Traditional	5.3	5.5	7.0	3.8	29.1	132.1
**Husband/partner educational status**						
None	7.4	11.1	22.5	51.4	102.7	212.7
Primary	16.9	25.7	38.5	52.1	49.8	127.8
Secondary+	52.2	51.2	59.3	−1.9	15.8	13.6
**Women educational status**						
None	9.3	21.5	35.1	13.1	63.3	277
Primary	23.6	21.5	35.7	−9.3	66.1	51.3
Secondary+	72.4	20.8	36.4	−71.3	75.5	−49.6
**Household Wealth quantile**						
Poor	5.2	9.6	21.7	86.5	126.0	317.3
Middle	9.3	15.5	33.1	66.7	112.9	254.8
Rich	33.4	40.4	56.2	21.3	39.1	68.6
**Residence**						
Rural	8.94	13.88	27.80	55.3	100.1	211.0
Urban	61.74	55.56	66.13	−10.0	19.0	7.1
**Place of Delivery**						
Home	9.9	13.9	19.7	39.4	41.7	98.0
H institution	68.6	62.8	61.7	−8.5	−1.8	−10.1
**Birth order**						
**1**	26.7	33.7	46.8	26.2	38.9	75.7
2–3	21.7	24.4	40.3	12.4	65.2	85.7
4–5	11.6	17.1	31.2	47.4	82.5	167.0
6+	9.4	13.3	23.8	41.5	79.7	154.3
**Trimester of ANC visit**						
1st trimester	78.0	68.8	73.3	−13.9	6.5	−6.0
2nd trimester	54.2	45.9	49.0	−15.3	6.7	−9.6
3rd trimester	9.8	8.3	5.7	−15.3	−31.3	−41.8
**Media exposure**						
Exposed	30.5	31.3	53.7	2.6	71.6	76.1
Non-exposed	8.0	9.7	25.5	8.8	161.9	218.8
**Fertility preference**						
Wants soon	17.0	21.2	35.3	24.7	66.5	107.6
Wants later	12.9	22.6	20.6	75.2	−8.8	59.7
Undecided	15.1	17.9	28.9	18.5	62.0	91.4
Wants no more	16.6	22.4	36.4	35.0	62.5	119.3
**Parity**						
1	26.6	33.7	46.8	26.3	38.9	75.1
2–3	21.7	24.4	40.3	12.4	65.2	85.7
4–5	11.6	17.1	31.2	47.4	82.5	167.0
6+	9.7	13.3	23.8	36.1	79.7	145.4
**History of abortion**						
Yes	25.1	22.6	40.0	−10.0	77.0	59.4
No	16.4	21.4	35.0	29.9	63.6	113.4
**History of stillbirth**						
Yes	16.4	21.4	35.0	30.0	63.6	113.4
No	25.1	22.6	40.0	−10.0	77.0	59.4
**Pregnancy Complication**						
Yes	44.7	65.1	65.1	46.0	−0.2	45.6
No	66.0	43.0	44.4	−34.8	3.3	−32.7

Acceptable ANC: attending at least four ANC visits, received at least one dose of tetanus toxoid (TT) injections and consumed 100 iron-folic acids (IFA) tablets/syrup during the last pregnancy

### Decomposition analysis of an acceptable antenatal care coverage

#### Difference due to characteristics (endowments)

The decomposition analysis revealed that about 29% of the overall percentage change in an acceptable ANC visit was due to a difference in characteristics (compositional factors). Among compositional factors, a significant contribution to the change in acceptable ANC visit was associated with the husband’s education, religion, wealth status, residence, and place of delivery (Table 3).

**Table 3:** Decomposition analysis of an acceptable antenatal care visit (ANC4+) among women who gave birth in Ethiopia, Ethiopian Demographic and health Survey 2005–2016

Variables	Difference due to characteristics (E)		Difference due to coefficient (C)	
	Coeff (95% CI)	Pct.	Coeff (95%CI)	Pct.
**Religion**				
*Traditional*	1	1	1	1
*Catholic*	− 0.00093(−.001302, −.000553)**	− 0.056	0.0057(.0026074 .0087822) ***	3.450
*Protestant*	0.006701(.004938, .008469)**	4.059	0.0966(.055246 .13786)* **	58.485
*Muslim*	0.0503(.036788, .063833)**	30.48	0.1452(.076146 .21423)* **	87.947
*Orthodox*	−0.0879(− 0.10989 -0.06588)**	−53.237	0.2089(.1224 .29556)* **	126.59
**Women education**				
No education	1	1	1	1
Primary	0.00078(−.00089755 .0010526)	0.047	−0.00327(−.010151 .0036074)	− 1.982
Secondary+	.000178(−.0012603 .0016159)	0.108	− 0.00342(−.0060527–.000773)**	−2.067
**Husband education**				
No education	1	1	1	1
Primary	0.00311(.00031283,.0058987) *	1.881	−0.00493(−.0169 .0070143)	−2.994
Secondary+	0.00021(−.0000278 .00043332)	0.123	−0.00670(−.012203 -.001201)**	−4.060
**Birth order**				
1	1	1	1	1
2–3	−0.000075(−.0003556 .0002069)	−.045	0.00168(−.0003556 .00020694)	−0.045
4–5	−0.00012 (−.0002856 .0000530)	−0.075	0.01629(.0041248 .028457)**	9.868
6+	0.00041(−.00067737 .0014853)	0.245	0.00636(−.00830 .020913)	3.820
**Media exposure to ANC visit**				
No	1	1	1	1
Yes	−0.00134(−.002972 .000313)	−0.805	− 0.0066(−.019636 .0063422)	−4.026
**Wealth status**				
Poor	1	1	1	1
Middle	−0.00103(−.00201–.00003583)*	−0.623	− 0.00457(−.015329 .0061896)	−2.768
Rich	−0.00256(−.00388–.0012548)***	−1.556	−0.0072(−.021949 .007582)	−4.351
**Residence**				
Urban	1		1	1
Rural	0.0035(.0003644 .0067244)* *	2.147	0.0764(.027658 .12512)* **	46.272
**Place of delivery**				
Home	1	1	1	1
H/Institution	0.0424(.033884 .050841)***	25.66	0.00127(−.0010716 .0036056)	0.768
**History of still Birth**				
No	1	1	1	1
Yes	0.00033(−.0020363 .0026985)	0.201	0.0348(−.037367 .10691)	21.062
**Constants**			−0.4367(−.66039 -.21296)***	− 264.51
**Overall**	.048614 (.038313 .058914)***	29.45	0.1165(.095227 0.13772)* *	70.55

Acceptable ANC: attending at least four ANC visits, received at least one dose of tetanus toxoid (TT) injections and consumed 100 iron-folic acids (IFA) tablets/syrup during the last pregnancy

*Pct* percentage contribution

*significant at 0.05

** significant at 0.01

*** significant at < 0.001

**Fig. 3:**
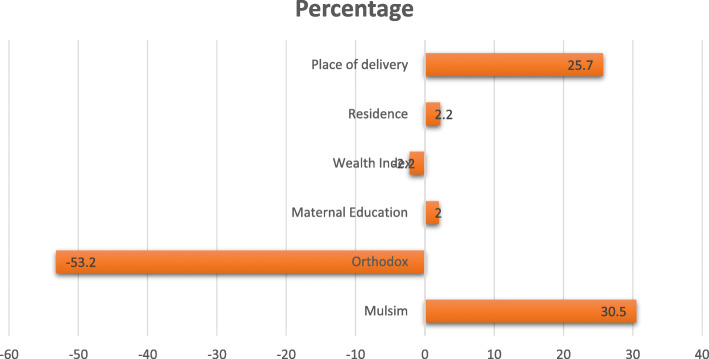
Contributions of change in the distribution ‘compositional effect’ of the determinants of ANC4+ in Ethiopia

A husband who attains primary school was an important contributor to the increment of acceptable ANC visits. The proportion of husbands who attain primary school increases from 21 to 32% in the last decades, with an important compositional contribution to the improvements of acceptable ANC visit by 2% (Fig. 3).

Religion is a significant contributor to the improvements of an acceptable ANC visit. The proportion of Muslim followers who visit acceptable ANC was doubled in the last decades, 13% in 2005, and 27% in 2016 (Table 2), with a significant compositional contributor in the improvements of acceptable ANC visit by 30% (Table 3).

Similarly, the proportion of women who deliver in health institutions increases from 5 to 27% in the last decades, with a significant compositional contributor in the improvements of acceptable ANC visit by 26% (Table 3).

Also, the residence is a significant compositional contributor to the improvements of an acceptable ANC visit. The percentage of women who visit acceptable ANC residing in rural areas was tripled in the last decades, 9% in 2005, and 28% in 2016 Table 2, with a significant compositional contributor for the increment of acceptable ANC visit by 2% (Table 3).

#### Difference due to effect of coefficients (C)

After controlling the role of compositional changes, 71% of improvements were due to behavioral change towards acceptable ANC visit controlling the roll of change in compositional characteristics (Table 3). Factors including Educational status, birth order, religion, and residence associated with a significant effect of coefficient contribution to the change in an acceptable ANC visit. Controlling the role of compositional changes, women‘s education and husband education (completed secondary and higher) had a significant contribution to the increase in an acceptable ANC visits by 2 and 4% respectively over the last decades.

Controlling the role of compositional changes, compared with followers of the Catholic religion, followers of other religions, especially Orthodox Christian religion showed significantly associated with the contribution to the increase in an acceptable ANC visit over a decade. The effect of religion becomes more important over time (Table 3). Further, the behavioral change in the rural population leads to the improvements of acceptable ANC visit by 46% controlling the effects of change in compositional factors (Table 3).

## Discussion

This study aimed to examine the trends and the major factors associated whether positively or negatively contributing to the change in acceptable ANC visit over the last decades.

ANC4+ visit was increased substantially over the last 10 years especially in the second survey period 2011–2016 i.e. by 14%. This might be attributed to the demanding efforts of the government to create awareness for the community about the significance of ANC service to meet the millennium development goal (MDG) via the health sector development plans [25].

All most two-third of the overall change in an acceptable ANC visit was due to differences in coefficients (C), implied that a significant contribution of the change arises when changing population behaviors via a significant explanatory variable. In this study religion is a significant contribution to the improvements of an acceptable ANC visit, meaning that there was a cultural belief that everyone is done on the will of God, the help of health professionals on birth, and pregnancy-related consultations are refused in most rural areas in Ethiopia. However, the government of Ethiopia had been done a pertinent awareness creation for the rural communities by enhancing community involvement and empowering through participation to identify needs, suggested solutions, implementation, and follow up activities to solve such challenges through the launch of health extension workers [26, 27].

Women’s attainment of secondary and above education exhibited a significant contribution to the improvements of an acceptable ANC visit consistent to a study done in Sub-Saharan Africa systematic review and meta-analysis [28]. Ethiopia has been worked to achieve the millennium development goal to advocate women’s educational attainment and lunched the growth and transformation plan I (GTP I) [20, 29]. Therefore, the compositional increase in both husband and women’s education in the last decade had a positive contribution to the improvements of ANC4+ service.

Similarly, a compositional change in husband primary education attainment has a significant contribution to the improvements of an acceptable ANC visit consistent in a study [20, 30]. Meaning that educated husband had a positive attitude towards the importance of frequent ANC visit, this leads to make a strong decision and support to use frequent ANC service for safe birth outcomes.

In terms of wealth status, a compositional change in a middle and rich person over time has a significant contribution to the improvements of an acceptable ANC visit over the last 10 years. This might be the improvement of the economy helps to advance health care utilization and able to afford medical and non-medical costs associated with ANC service during pregnancy [2, 5, 19, 31, 32]. Thus, lack of financial access is a barrier to use an acceptable ANC service by pregnant women; it limits the number of ANC visits or even initiates ANC late during pregnancy [19].

The proportion of rural women who use ANC4+ is increased from 9% in 2005 to 28% in 2016, with an overall decomposition change in coefficients/ change in behavior of women by 46% in the last 10 years. Even though, the proportion of urban women an acceptable ANC use was 66% in 2016, even far exceeds rural proportion in the same year. However, the high progress has been observed in the rural areas where most of the population lives previous studies documented similarly [32, 33]. This might be due to the execution of the Health extension workers [34, 35] and expansion of primary health care units in the last decade via rehabilitation and advancing of the existing health facilities as well as the building of new facilities i.e. the number of a health post and health centers in 2005 was 6, 191and 668 while in 2013 this number was increased to 16, 045 and 3, 245 respectively [27, 34–37]. Also, the provision of health insurance, free medical costs, improvements of human resource and road construction in each district might be contributed to the improvements of ANC4+ for pregnant women in Ethiopia.

Also, an astonishing finding was obtained in this analysis is in the effect of religion both the composition of characteristics (Endowment) and coefficients/behavioral change in women. The change in coefficients/ behavior among orthodox Christians contributed to the improvements of an acceptable ANC visits during pregnancy in line with a study conducted in Sub-Saharan Africa [28]. However, there is no supportive evidence on the reason for differences among religions.

The strength of this study was, the study was done on large data set representing the whole country, and thus findings were based on adequate statistical power. Second, calculations were utilized after the data were weighted for sampling probabilities and non-response. Complex sampling procedures were also considered during testing of statistical significance. Third, analytic techniques such as decomposition analysis were done to understand the source of change in acceptable ANC visits.

The study tries to address important findings to support an acceptable ANC visits in Ethiopia, however not without limitation which may affect the conclusions of our findings. As the data were cross-sectional surveys likely to prone to recall bias and social desirability bias. During decomposition analysis, important variables such as women’s decision-making capacity, type investigation during ANC visit, maternal health service and quality, maternal medical and obstetric condition; variables like diabetes mellitus, hypertension, HIV/AIDS, heart failure, renal disease and attitude towards ANC were not addressed in this study because these variables were not available. Further research is needed including alternative methodology to the decomposition analysis.

## Conclusion and recommendation

An acceptable ANC visit among women has been slightly increased over the last 10 years in Ethiopia. Nearly, one-third of the overall change in acceptable ANC visit over the last 10 years was due to the difference in characteristics of the population between 2005 and 2016 in Ethiopia. The compositional change in religion, husband educational attainment, residence, and wealth status are the potential factors for the improvements of acceptable ANC visit in Ethiopia. Also, almost two-thirds of the improvement in acceptable ANC visit was relay on change in coefficients/behavior of pregnant women towards acceptable ANC visit. Factors contributed to the change in coefficients of acceptable ANC visits are residence, religion, and educational attainments.

Public intervention should continue to enhance the ANC program and further advancing of health care facilities should be done in rural communities to maintain further improvements of acceptable ANC visit. It is mandatory advancing the education of the young population to empower girls and develop a positive attitude towards ANC visits during pregnancy.

## Data Availability

The data was available from the corresponding author and we can provide upon request.

## Abbreviations

AIDS: Acquired immunodeficiency syndrome
ANC: Antenatal care
EAs: Enumeration areas
EDHS: Ethiopia Demographic and Health Survey
EPHC: Ethiopian Population and Housing Census
CDC: Center of Disease Control
EHNRI: Ethiopian DHS obtained Ethical clearance from Ethiopian Health Nutrition and Research institute
IRB: Institutional Review Board
HIV: Human immunodeficiency virus
NGO: Non-governmental organizations
SNNP: Southern nations, nationalities, and people’s region
MDG: Millennium development goal
SSA: Sub-Saharan Africa
SDG: Sustainable development goal
TBA: Traditional birth attendance
TT: Tetanus toxoid
WHO: World Health Organization
